# Small bowel adenocarcinomas with favorable prognoses by radical resection and adjuvant chemotherapy: a case series of five cases

**DOI:** 10.1186/s40792-023-01771-z

**Published:** 2023-10-30

**Authors:** Yuri Nishioka, Yasunori Matsumoto, Shunsuke Imanishi, Satoshi Endo, Takeshi Toyozumi, Yoshihiro Kurata, Takuma Sasaki, Gaku Ohira, Koichi Hayano, Hisahiro Matsubara

**Affiliations:** Department of Frontier Surgery, Chiba University Graduate School of Medicine, 1-8-1Inohana, Chuo-ku, Chiba-shi, Chiba 260-8670 Japan

**Keywords:** Small bowel adenocarcinoma, Jejunal adenocarcinoma, Adjuvant chemotherapy, Surgery

## Abstract

**Background:**

Small bowel cancer is very rare, accounting for less than 5% of all gastrointestinal cancers, and small bowel adenocarcinoma accounts for approximately 40% of all small bowel cancers. Small bowel adenocarcinoma is often found in advanced stages, with only 40–65% of cases being curatively resectable. The prognosis is poor, with a 5-year survival rate of 14–33% for all patients and 40–60% for those who are curatively resectable. In Japan, practice guidelines for duodenal cancer were instituted in 2021. However, evidence-based standard treatments have not been established for jejunal and ileal cancers. In particular, chemotherapeutic options are limited, and there are only a few reports on multidisciplinary treatments, including adjuvant chemotherapy.

**Case presentation:**

We report five cases of jejunal or ileal lesions that were treated with adjuvant chemotherapy after radical resection. Three patients were male and two were female, with a median age of 67 years. Tumor localization was observed in the jejunum in all cases. Clinical staging was as follows: stage IIIA in two cases and stage IIIB in three cases. Laparotomy was then performed in all cases, employing partial resection with lymph node dissection. Pathological staging in all cases was as follows: stage IIB in two cases, stage IIIA in one case, and stage IIIB in two cases. In all cases, the regimen for adjuvant chemotherapy was selected based on the colorectal cancer guidelines. No serious complications arose from adjuvant therapy; however, adverse events occurred in patients receiving multi-agent chemotherapy. No recurrence was observed in any of the cases, and all the patients survived, with a median survival time of 32 months. As a representative case, we present a case of adjuvant chemotherapy for jejunal adenocarcinoma staged as pT3N2M0, pStage IIIB, with no recurrence at 32 months postoperatively.

**Conclusions:**

In general, favorable outcomes were achieved with adjuvant therapy applied in accordance with the criteria for colorectal cancer. These favorable outcomes suggest that it is necessary to identify the risk factors and indications for adjuvant therapy, specifically for small bowel adenocarcinoma.

## Introduction

Small bowel cancer is very rare, accounting for less than 5% of all gastrointestinal cancers, despite the fact that the small intestine accounts for 75% of the digestive tract’s length and 90% of the tract’s mucosal surface area. Small bowel adenocarcinoma (SBA) accounts for approximately 40% of all small bowel cancers [1]. SBA is often found in advanced stages, with only 40–65% of cases being curatively resectable [2]. As there are no specific clinical symptoms, diagnosis is usually delayed. Most cases are found in advanced stages, with 35% of patients having synchronous metastases and 39% having tumors with lymph node invasion [2]. The prognosis is poor, with a 5-year survival rate of 14–33% for all patients and 40–60% for those who are curatively resectable [1, 2]. In Japan, practice guidelines for duodenal cancer were instituted in 2021. However, evidence-based standard treatments have not been established for jejunal and ileal cancers. In particular, chemotherapeutic options are limited, and there are only a few reports on multidisciplinary treatments, including adjuvant chemotherapy. Herein, we report five cases of adenocarcinoma of the jejunum and ileum treated with adjuvant chemotherapy after radical resection.

## Case presentation

### Patients and methods

Twenty-nine patients who underwent surgery for SBA, including duodenal adenocarcinoma, at Chiba University Hospital between January 2012 and December 2021 were retrospectively reviewed. Patients with jejunal or ileal lesions who received adjuvant chemotherapy after radical resection were included in this study. Data on demographic details; clinical history; preoperative evaluation, including blood tests and imaging studies; treatment details; histopathological examination results; clinical course; and survival follow-up were extracted from the patients' medical records. Tumor, node, and metastasis (TNM) classification and staging were performed according to the 8th edition of the Union for International Cancer Control Staging System (UICC). Histological classification was performed according to the Japanese Classification of Colorectal Carcinomas. Postoperative complications were evaluated using the Clavien–Dindo classification. Chemotherapy-related adverse events were evaluated according to the Common Terminology Criteria for Adverse Events (CTCAE) version 5.0.

### Results

**Fig. 1:**
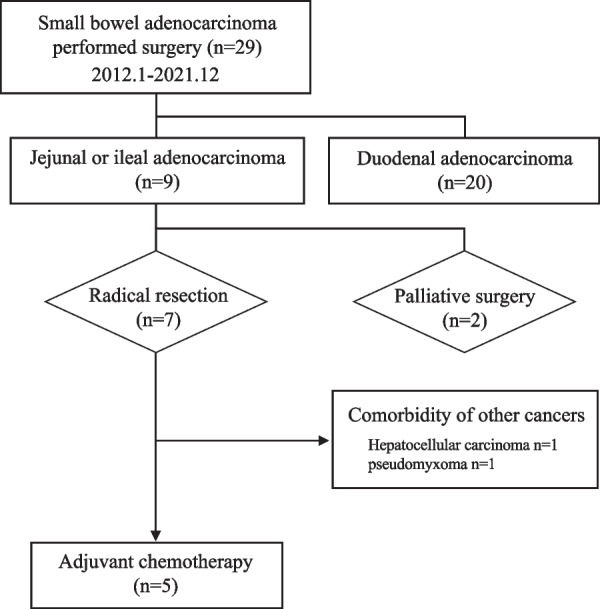
Flow diagram of patient selection

**Fig. 2:**
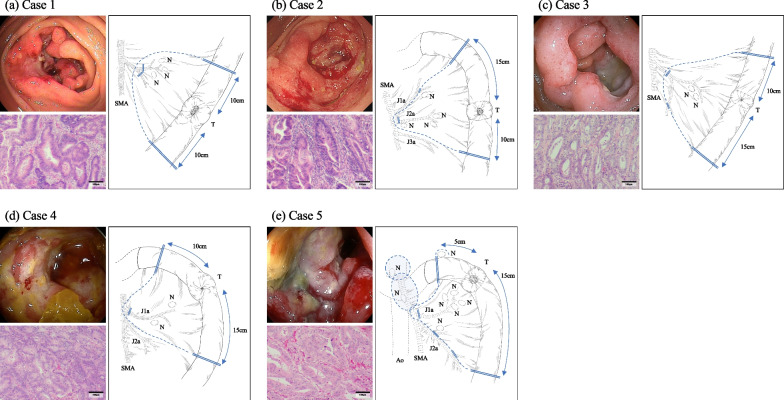
Imagings and findings of each patient. **a**–**e** correspond, respectively, to Cases 1–5. In each case, the upper left displays the preoperative endoscopic findings, the right shows the operative findings, and the lower left presents the histopathological findings from resected specimens. In the operative findings, lymph node dissection areas are indicated by dashed lines, and vascular and bowel resection lines are indicated by double lines. Histopathological findings were evaluated using hematoxylin–eosin (HE) stained images. T: tumor; N: enlarged lymph nodes on surgery; Ao: abdominal aorta; SMA: superior mesenteric artery; J1a: first branch of the jejunal artery; J2a: second branch of the jejunal artery; J3a: third branch of the jejunal artery

Of the 29 SBA cases, 20 had duodenal involvement and nine had jejunal or ileal involvement. Radical surgery was performed in seven patients and palliative surgery in two patients. Five patients, excluding two patients in whom other cancers were present, underwent adjuvant chemotherapy according to the criteria for colorectal cancer (CRC) (Fig. 1). The endoscopic findings, histopathological results from the resected specimens, and surgical resection areas in each patient treated with adjuvant therapy are shown in Fig. 2.

Patient backgrounds are shown in Table 1. Three patients were male and two were female, with a median age of 67 years (range 45–75). Tumor localization was observed in the jejunum in all the cases, with three cases on the proximal side and two cases on the distal side. Four patients experienced subjective symptoms on their first hospital visit, including abdominal pain, distension, nausea, and vomiting. Preoperative diagnoses were made using double-balloon endoscopy in two cases, contrast-enhanced computed tomography (CT) in two cases, and in one case, the tumor was discovered incidentally through positron emission tomography/computed tomography (PET–CT) imaging for a comorbidity. All patients were histologically diagnosed through preoperative endoscopic biopsy. One patient had increased carcinoembryonic antigen (CEA) levels preoperatively and three had elevated Carbohydrate antigen 19-9 (CA19-9) levels. All values decreased postoperatively. Clinical staging was as follows: stage IIIA in two cases and stage IIIB in three cases.

**Table 1:** Patient backgrounds

Case No	1	2	3	4	5
Age, years	45	54	71	67	75
Sex	Male	Male	Female	Female	Male
Tumor localization	Jejunum	Jejunum	Jejunum	Jejunum	Jejunum
Subjective symptoms	Abdominal pain	Abdominal pain, abdominal distension	Abdominal pain	Nausea, vomiting	None
Diagnostic opportunity	Oral double-balloon endoscopy	Oral double-balloon endoscopy	Contrast-enhanced CT	Contrast-enhanced CT	PET–CT
Preoperative histological diagnosis	Adenocarcinoma	Adenocarcinoma (tub2)	Adenocarcinoma (por)	Adenocarcinoma (tub2-1)	Adenocarcinoma (tub1-2, por)
Preoperative CEA, ng/mL (→ post*)	1.7	4.1	2.6	1.9	8.1 → 2.1
Preoperative CA19-9, U/mL (→ post*)	2.1	< 0.1	879.6 → 8.7	36.3 → 7.9	530.5 → 43.9
Clinical Stage (UICC 8th)	cT3, cN2, cM0, cStage IIIB	cT3, cN1, cM0, cStage IIIA	cT3, cN2, cM0, cStage IIIB	cT3, cN1, cM0, cStage IIIA	cT3, cN2, cM0, cStage IIIB

^*^One-year postoperative levels in cases with high preoperative levels

CT: computed tomography; PET–CT: positron emission tomography/computed tomography; tub1: Well-differentiated tubular adenocarcinoma; tub2: moderately differentiated tubular adenocarcinoma; por: poorly differentiated tubular adenocarcinoma; CEA: carcinoembryonic antigen; CA19-9: carbohydrate antigen 19-9

The surgical and histopathological findings are shown in Table 2. Partial resection with lymph node dissection was performed through laparotomy in all cases. Surgical margins ranged from 5 to 15 cm. The median operative time was 148 min (range 121–222), and the median blood loss was 85 g (range 30–390). None of the resultant postoperative complications were classified as Clavien–Dindo classification grade 3, and the median postoperative hospital stay was 12 days (range 6–14). All tumors were circumferential. Three tumors were type 2, and two were type 3, according to macroscopic classification. Pathologic features included poorly differentiated tubular adenocarcinoma in three cases. Pathological staging in all cases was as follows: stage IIB in two cases, stage IIIA in one case, and stage IIIB in two cases. The median number of dissected lymph nodes was 16 (range 7–37). Venous invasion was positive in four cases, lymphovascular invasion in two cases, and paraneural invasion in two cases. In all cases, resection margins were negative. Both cases of pStage II had high-risk stage II factors, as in CRC; one had pT4 and venous invasion, and the other had bowel obstruction, pT4, and paraneural invasion.

**Table 2:** Surgical and histopathological findings

Case No	1	2	3	4	5
Operative method	Partial jejunectomy, lymph node dissection	Partial jejunectomy, lymph node dissection	Partial jejunectomy, lymph node dissection	Partial jejunectomy, lymph node dissection	Partial jejunectomy, lymph node dissection
Distance from Treitz ligament	110 cm	15 cm	110 cm	10 cm	5 cm
Surgical margin (proximal, distal)	10 cm, 10 cm	15 cm, 10 cm	10 cm, 15 cm	10 cm, 15 cm	5 cm, 15 cm
Operative time	148 min	121 min	139 min	178 min	222 min
Blood loss	190 g	30 g	40 g	85 g	390 g
Postoperative complications*	None	None	None	None	None
Postoperative hospital stay	6 days	9 days	14 days	12 days	14 days
Tumor size**, mm	63 × 45, circumferential	49 × 29, circumferential	44 × 19, circumferential	75 × 44, circumferential	50 × 30, circumferential
Macroscopic classification	Type 2	Type 3	Type 2	Type 3	Type 2
Pathological feature	tub2 > tub1	tub2	por	muc, tub2, tub1, por	por > > tub1 > tub2
Pathological Stage (UICC 8th)	pT4, pN0 (0/37), pM0, pStage IIB	pT4, pN0 (0/12), pM0, pStage IIB	pT3, pN2 (4/7), pM0, pStage IIIB	pT3, pN1 (1/16), pM0, pStage IIIA	pT4, pN2 (8/16), pM0, pStage IIIB
Venous invasion	Positive	Negative	Positive	Positive	Positive
Lymphovascular invasion	Negative	Negative	Positive	Negative	Positive
Paraneural invasion	N/A	Positive	Negative	Negative	Positive
Resection margin	Negative	Negative	Negative	Negative	Negative
Factors of high-risk Stage II	pT4, Venous invasion	Obstruction, pT4, Paraneural invasion	–	–	–

^*^Grade 3 above in Clavien–Dindo classification. **(Circumference diameter) × (Lesion length)

tub1: well-differentiated tubular adenocarcinoma; tub2: moderately differentiated tubular adenocarcinoma; por: poorly differentiated tubular adenocarcinoma; muc: mucinous adenocarcinoma; N/A: not available

The course of adjuvant therapy and patient prognoses are shown in Table 3. In all cases, the regimen was selected based on the CRC guidelines. Three patients experienced adverse events during chemotherapy, including liver dysfunction, neutropenia, and oxaliplatin allergy. One patient had a regimen change, and two patients discontinued chemotherapy because of adverse events that occurred during the last course of chemotherapy. At the time of this report, no recurrence was observed in any of the patients, and all patients were alive with a median survival time of 32 months (range 22–121).

**Table 3:** Course of adjuvant therapy and patient prognoses

Case No	1	2	3	4	5
Adjuvant chemotherapy	S-1	UFT + LV	CAPOX → S-1	CAPOX	FOLFOX
Period of adjuvant chemotherapy	12 months	5 months	6 months	4 months	5 months
Adverse events of chemotherapy (CTCAE Grade)	None	None	Liver dysfunction (Grade 2)	Neutropenia (Grade 3)	Allergy to oxaliplatin (Grade 1)
Relapse-free survival period	121 months	47 months	32 months	24 months	22 months

LV: Leucovorin; CAPOX: Capecitabine and Oxaliplatin; FOLFOX: Fluorouracil, Leucovorin, and Oxaliplatin; CTCAE: Common Terminology Criteria for Adverse Events

#### A representative case (Case 3)

The patient was a 71-year-old woman who complained of abdominal pain. Blood analysis revealed a low hemoglobin level (10.4 g/dL) and a high serum CA19-9 level (879.6 U/mL). Contrast-enhanced CT revealed a small bowel tumor and the presence of enlarged lymph nodes in the surrounding area. Oral double-balloon endoscopy indicated a full circumferential type 2 tumor located 110 cm from the Treitz ligament; however, due to stenosis, the endoscope could not pass through. The pathologic diagnosis based on biopsy was a poorly differentiated tubular adenocarcinoma. PET–CT showed hyperaccumulation of fluorine-18 deoxyglucose in the primary tumor and the enlarged lymph nodes, with no other foci noted. Based on these findings, the preoperative diagnosis was jejunal adenocarcinoma, staged as cT3N2M0, cStage IIIB.

Surgery included a partial jejunectomy and lymph node dissection performed via laparotomy. The macroscopic examination of the resected specimen revealed a full circumferential type 2 tumor. The histopathological diagnosis was a poorly differentiated tubular adenocarcinoma, pT3N2M0, and the tumor was classified as pStage IIIB.

In accordance with the criteria for colorectal cancer, postoperative adjuvant chemotherapy was planned and explained sufficient information to the patient. The patient underwent treatment with capecitabine and oxaliplatin (CAPOX), but it was discontinued after 3 courses due to Grade 3 liver dysfunction, and the treatment was switched to S-1. There were no adverse events after the change in the treatment regimen. The patient is currently alive without any signs of recurrence, having passed 32 months since the surgery and 24 months since the completion of chemotherapy.

## Discussion

In this report, we described five cases of jejunal adenocarcinoma treated with adjuvant chemotherapy after curative resection that resulted in recurrence-free survival. In general, SBA has a poor prognosis, and its prognosis after curative resection is not favorable. Moreover, the pattern of recurrence after curative resection frequently involves distant metastasis [2–4]. These findings suggest the importance of adjuvant systemic therapy in patients with a high risk of recurrence. However, there are few reports on adjuvant therapy for resectable SBA, and the regimens and outcomes are controversial. Ecker et al. [5] reported that adjuvant chemotherapy significantly improved overall survival compared with surgery alone in stage III, although there was no statistically significant difference between these two therapies in stage II. In contrast, another study found no significant differences in disease-free survival and overall survival between patients treated with and without adjuvant therapy, including radiotherapy [6]. In Japan, a phase III clinical trial (JCOG1502C; J-BALLAD trial) is currently ongoing to confirm the superiority of postoperative CAPOX over surgery alone in stages I–III SBA [7], and the results are expected soon.

Poor prognostic factors for SBA include pT4 tumors, poorly differentiated tumors, positive resection margins, and a metastatic lymph node ratio of ≥ 10% [3, 5, 6, 8]. However, the indications for adjuvant therapy are not specific to SBA and frequently follow the guidelines for colorectal or gastric cancer. In CRC, adjuvant chemotherapy is indicated for patients with stage III and high-risk stage II patients. High-risk stage II is defined by the presence of at least one of the following findings: pT4, poorly differentiated or undifferentiated cancer, vascular invasion, paraneural invasion, bowel obstruction or perforation as the initial presentation, or < 12 lymph nodes dissected [9]. Overman et al. [10] reported that cancer-related mortality was significantly reduced with an increased number of lymph nodes dissected in stages I–III SBA. Furthermore, in their study, the 5-year disease-specific survival rate decreased with seven or fewer dissected lymph nodes in stage II disease. According to Nicholl et al. [11], in patients with stage II jejunal and ileal adenocarcinomas, the 5-year survival rate increases when 10 or more lymph nodes are resected. Such an association between the number of dissected lymph nodes and prognosis may be an indicator for adjuvant therapy in SBA, similar to its role in CRC.

In our study, adjuvant therapy was administered according to indications and regimens for CRC, achieving successful results. The reasons for adopting the strategy of CRC are that the jejunum and ileum are embryologically similar to the right colon. Drugs were selected from the standard drugs in colorectal cancer at the time, considering the occurrence of adverse events, such as peripheral neuropathy, and basically, a combination/noncombination regimen of fluoropyrimidine and oxaliplatin was used. Although outcomes of our cases seem favorable considering the survival rates in previously reported resected cases, it is necessary to identify the risk factors and indications for adjuvant therapy, specifically for SBA. As a limitation of this study, it should be noted that there is a small number of cases, and it is unable to directly assess the additional effects of adjuvant therapy due to the absence of a comparison with surgical resection alone. In addition, this study is also limited in that some cases had a short observation period, and long-term prognosis cannot be evaluated. However, with the median time to recurrence of jejunal adenocarcinoma reported at 11.5 months [12], our cases are considered to have a relatively good postoperative outcome. In terms of safety, there were no serious complications from the adjuvant therapy; however, adverse events occurred in patients receiving multi-agent chemotherapy. Accumulating evidence regarding the appropriate indications and treatment intensity is required.

## Conclusions

We were able to successfully administer a relatively safe adjuvant chemotherapy regimen for advanced SBA after curative resection. Although generally favorable outcomes have been achieved using the same criteria for CRC, it is desirable to develop indications for adjuvant chemotherapy tailored to SBA.

## Data Availability

The data that support the findings of this study are available from the corresponding author upon reasonable request.

## Abbreviations

SBA: Small bowel adenocarcinoma
TNM: Tumor, node, and metastasis
UICC: Union for International Cancer Control Staging System
CTCAE: Common Terminology Criteria for Adverse Events
CRC: Colorectal cancer
CT: Computed tomography
PET–CT: Positron emission tomography/computed tomography
CEA: Carcinoembryonic antigen
CA19-9: Carbohydrate antigen 19-9
CAPOX: Capecitabine and oxaliplatin
